# No evidence that kin selection increases the honesty of begging signals in birds

**DOI:** 10.1002/evl3.18

**Published:** 2017-08-01

**Authors:** Kat Bebbington, Sjouke A. Kingma

**Affiliations:** ^1^ School of Biological Sciences University of East Anglia Norwich Research Park Norwich NR4 7TJ UK; ^2^ Behavioural & Physiological Ecology, GELIFES University of Groningen 9700CC Groningen The Netherlands

**Keywords:** Comparative studies, competition, kin selection, signaling

## Abstract

Providing plausible mechanisms to explain variation in the honesty of information communicated through offspring begging signals is fundamental to our understanding of parent–offspring conflict and the evolution of family life. A recently published research article used comparative analyses to investigate two long‐standing hypotheses that may explain the evolution of begging behavior. The results suggested that direct competition between offspring for parental resources decreases begging honesty, whereas indirect, kin‐selected benefits gained through saving parental resources for the production of future siblings increase begging honesty. However, we feel that evidence for a role of kin selection in this context is still missing. We present a combination of arguments and empirical tests to outline alternative sources of interspecific variation in offspring begging levels and discuss avenues for further research that can bring us closer to a complete understanding of the evolution of offspring signaling.

## A Short Introduction to Offspring Begging Signals

Across a diverse range of taxa, offspring direct behaviorally complex begging displays toward caregiving parents. The function and evolution of such behavior has intrigued biologists for decades, spawning a myriad of different explanatory hypotheses that make diverse assumptions about the balance of power between parents and their offspring (Royle et al. 2002a), the reliability of information that begging signals convey to parents (Kilner and Johnstone 1997) and the roles of kin selection and competition among offspring (Trivers 1972).

If parents make active choices about how to partition resources between their offspring, there are two scenarios where we can expect offspring begging to be an honest signal about the state of the offspring. First, if the cost of expressing a begging signal outweighs the marginal fitness gained from successfully securing parental resources, begging signals should honestly reflect the offspring's marginal fitness gain per unit of additional parental investment (Godfray 1995). Second, if there is a high risk that not all offspring survive to adulthood, begging signals should honestly reflect quality because individuals are selected to boast their own quality and/or parents are selected to preferentially invest in the most valuable offspring (Grafen 1990). However, the degree to which begging behaviors honestly reflect any such information seems to vary greatly between species (Mock et al. 2011). Although many studies support predictions of honest begging (e.g., Redondo and Castro 1992; Andrews and Smiseth 2013), others suggest that begging is a form of scramble competition for resources passively allocated by parents to the most conspicuous display (e.g., Smith and Montgomerie 1991; Parker et al. 2002). One possible source of this variation may be interspecific differences in the degree of evolutionary conflict within the family over the allocation of parental resources. Specifically, where high relatedness between family members means that their evolutionary interests in terms of resource allocation are more aligned (Trivers 1974), honesty should prevail. In contrast, where evolutionary interests are less aligned, for example when the direct fitness benefits of acquiring resources outweigh the benefits of sharing them with relatives, scramble competition and dishonesty should be more prevalent (Briskie et al. 1994).

## Drivers of Honest Begging Signals: A Recent Case Study

In a recent comparative analysis across avian taxa, Caro et al. (2016) aimed to explain interspecific variation in honesty of begging signals in relation to variation in conflict between family members over the allocation of parental care. Caro et al. (2016) first tested the hypothesis that begging honesty decreases with increasing competition for parental resources (Mock and Parker 1997). They showed convincing evidence that the correlation between begging and some measured component of offspring “need” (such as hunger levels) becomes weaker with the presence and increasing number of siblings in both current and future broods. These interspecific patterns provide important validation for the hypothesis that offspring competition for limited resources selects for exaggerated, and thus dishonest, begging signals (Royle et al. 2002a). Moving onto a second hypothesis, Caro et al. (2016) aimed to test whether begging is more honest when relatedness to future offspring, and hence the inclusive fitness benefit of sharing parental resources, is higher (Trivers 1974). According to Caro et al.’s (2016) interpretations, the results they present appear to support this second hypothesis; in doing so, they may provide the first empirical evidence that relatedness between competitors can effectively reduce parent–offspring conflict and offer one explanation for variation in begging honesty, one of the most widely debated phenomena in behavioral ecology. Below, we explain why it is premature to embrace the conclusions of Caro et al. (2016) as evidence for a role for kin selection in this context, and that multiple other processes may, alternatively or additionally, explain their findings.

## Estimating the Inclusive Fitness Value of Future Siblings

When an individual's parents can produce more offspring in the future, inclusive fitness benefits (i.e., the transfer of shared genes to future generations) may favor offspring who adopt strategies that facilitate the production of future siblings. Producing honest signals about current nutritional state to preserve parental resources (i.e., energy or food) for future broods (Trivers 1974) is one potential strategy. How then should we calculate expected inclusive fitness benefits from the perspective of current offspring? In their comparative analysis, Caro et al. (2016) suggested that relatively low inclusive fitness benefits arise when parents do not breed together to produce future broods, as is the case when (1) one or both of the parents die or (2) parents divorce. By combining these two measures, Caro et al. (2016) showed that offspring begging signals are less honest when parents have a lower likelihood to breed together in the future, which they interpreted as evidence that kin selection drives honesty of begging signals.

**Figure 1:**
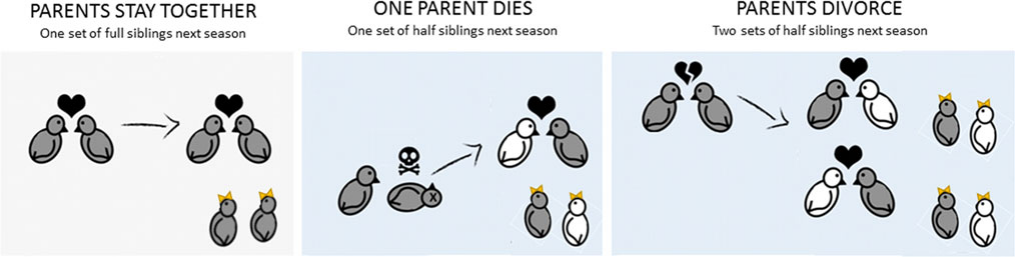
Modified from Caro et al. (2016). Kin selection predicts that offspring should be honest about their need when parents are likely to produce full siblings in future (left‐hand panel). If this is the case, the death of one parent (middle panel) should promote offspring dishonesty because of reduced relatedness to future offspring (relatedness = 1 × 0.25). However, we argue that divorce (right‐hand panel) does not promote dishonesty in this way because both parents will continue breeding and hence produce two sets of half‐siblings, which together have equal or even higher value than one set of full siblings (total relatedness **≥** 2 × 0.25 = 0.5).

Although we agree that the death of one parent indeed reduces future indirect benefits, it is incorrect to assume the same for divorce, and we therefore question whether the conclusion that kin selection underlies begging honesty is correct. As demonstrated in Figure 1, divorced parents will both produce half‐siblings with a total inclusive fitness value equal to that produced when they were together. In fact, the inclusive fitness benefits gained from offspring produced from divorced parents are (on average) greater than those from parents who remain together. In another recent comparative study, Culina et al. (2015) showed that divorce generally improves parents’ subsequent reproductive success, suggesting that offspring in species with high divorce rates should, on average, be under greater selection to beg honestly. Thus, combining divorce and mortality rates to generate a proxy for kin‐selected benefits may lead to an erroneous conclusion about whether kin selection may underlie variation in begging honesty.

**Figure 2:**
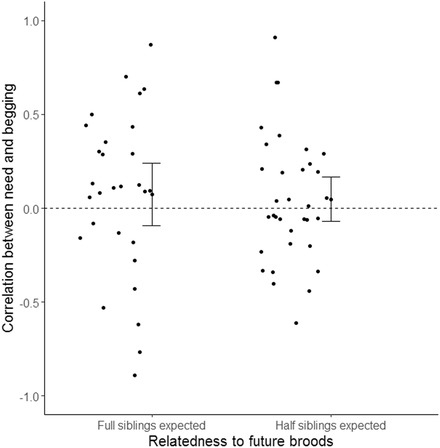
Relationship between begging honesty (measured as the correlation between begging intensity and need) and relatedness to future broods in 63 bird species. Full siblings are expected when there is <50% chance of at least one parent death before next year (34 species) and half siblings are expected when there is >50% chance of at least one parent death before next year (29 species). Raw data were plotted and error bars represent 95% CIs.

Having established that parental divorce may not necessarily reduce the kin‐selected incentives of current offspring to beg honestly, we retested the hypothesis that high inclusive fitness benefits of future offspring select for honest signaling, using an identical set of species and the same sample size as Caro et al. (2016). To provide a more accurate calculation of inclusive fitness benefits in terms of the likelihood of full siblings being produced in future, we disregarded divorce rates and only used the likelihood of both parents surviving to reproduce next season. Data were obtained from Caro et al. (2016), and phylogenetic generalized least square (PGLS) analyses were implemented in the Caper package (Orme et al. 2013) in R 3.3.0 (R Development Core Team 2016). We accounted for phylogenetic uncertainty by applying the models to a set of 100 phylogenetic trees (using the Hackett backbone with all species) obtained from http://www.birdtree.org (Jetz et al. 2012). In contrast to the results reported by Caro et al. (2016), we found no difference in the correlation between begging and need (i.e., begging honesty) according to whether the chance of both parents surviving was greater than 50% (PGLS: *β* ± SE = –0.026 ± 0.098, *t*_61_ = –0.271, *P* = 0.787, Fig. 2). We also were unable to support the conclusion of Caro et al. (2016) if, instead of using this classification, we tested the effect of the absolute probability that both parents survive (range = 2–88%) (PGLS: *β* ± SE = 0.073 ± 0.2071, *t*_,61_ = 0.351 *P* = 0.727).

As we outline above and in Figure 1, variation in divorce rates is unlikely to reduce future inclusive fitness. Because divorce accounted for on average (±SE) 49 ± 4% of Caro et al.’s measure of “likelihood that pairs did not breed together the following year” (44 species, range = 0–99%), it is perhaps not surprising that when we omit divorce rates from the equation we cannot support the conclusion that kin selection plays a role in honest begging. However, an important question remains: how then can we explain the intriguing relationship found by Caro et al. (2016) that begging is more honest if pairs have a low likelihood to breed again together the following year? In the final section of this article, we outline a series of arguments that provide both potential explanations for the results presented in Caro et al. (2016) and exciting avenues for further research on the evolution of offspring begging signals more generally.

## Beyond Kin Selection: Explaining Variation in Offspring Begging Honesty

As outlined above, scramble competition for limited parental resources may be an important mechanism that decreases signal honesty (Royle et al. 2002a); in line with this, Caro et al. (2016) show that begging signals are less honest in the face of competition with coexisting offspring. We propose that the relationship between begging honesty and pair bond duration (or the “likelihood that parents reproduce together in future”) can also be explained in terms of offspring competition. Below, we present three potential mechanisms by which offspring competition could drive this relationship, one of which we were able to test using Caro et al.’s (2016) dataset. Although we explicitly consider variation in offspring begging in the context of the relationship between honesty and pair bond duration, it is important to note that these alternative hypotheses are entirely speculative at this point. Nonetheless, we believe that these and other mechanisms are interesting to consider in relation to offspring begging signals more generally and may stimulate future work.

(1) Pair bond duration is associated with clutch size and offspring competition

**Figure 3:**
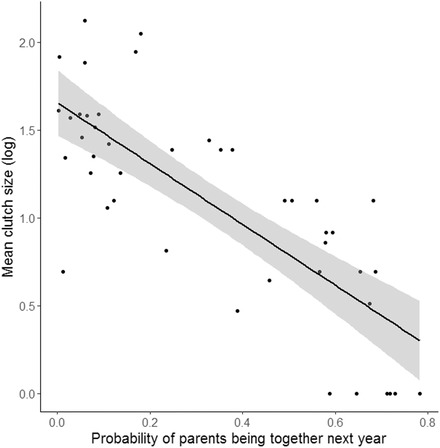
Relationship between mean clutch size (log transformed) and the probability of parents reproducing together in the next year across 44 bird species. Untransformed raw data were plotted (with the regression line through the raw data) and shaded areas represent 95% CIs.

If offspring begging honesty is related to competition for limited resources, species where sibling competition is more intense are expected to be less honest. Because individuals with relatively short lifespans and hence short pair bonds produce a large number of offspring in each reproductive attempt (Charnov and Krebs 1974; Martin 2002), offspring competition might be higher within broods of species with short pair bonds. Using the dataset from Caro et al. (2016), we used PGLS analyses (as described above) to test for a correlation between the likelihood that parents produce together in future and levels of current offspring competition in terms of clutch size. Although the amount of parental resources per offspring would give a truer measure of the degree of offspring competition than the absolute number of competitors (Mock et al. 2009), measuring clutch size at least captures some of the variation between offspring raised on their own (who by definition experience no direct competition) and those raised with siblings (where there is at least potential for competition). We found that species where parents have a higher probability of breeding together in the following year (calculated as: [(survival probability)^2^ × (one‐divorce rate)] produce broods of smaller size (log‐transformed; PGLS: *β* ± SE = –0.684 ± 0.122, *t*_42_ = –5.608, *P* < 0.001, Fig. 3), a pattern that is partly driven by the long pair bond duration in species that have only one offspring per brood (Fig. 3). This result suggests that the association between parental pair bond duration and begging honesty can, at least partly, be explained by the fact that competition in current broods of species with short pair bonds is higher. Further exploration of this pattern with a more accurate representation of offspring competition, such as the proportion of offspring that recruit per brood, the degree of asymmetry in offspring size, or the amount of parental provisioning, would be very useful to confirm this relationship.

(2) Parental divorce is linked to social mate competition

Among bird species, parental divorce rates are linked to extra‐pair paternity (Cezilly and Nager 1995) and mutual ornamentation (Kraaijeveld 2003; Botero and Rubenstein 2012); both these factors reflect the level of social competition for mates. If species with high divorce rates are characterized by high levels of mate competition, we can expect strong selection for competition‐related behavioral traits in such species. Because traits that increase reproductive success in adulthood (in this case, traits that increase competitive ability) will be present in the offspring of successful adults, we can predict that offspring in species with high divorce rates are more competitive and less likely to beg honestly. The idea that offspring behaviors may be influenced by selection for adult behaviors is not new (Kölliker et al. 2012), but perhaps revisiting models of offspring begging behavior in the light of parent–offspring coadaptation may reveal intriguing new patterns.

(3) Pair bond duration is associated with increased sexual conflict over care

A key principle of life‐history theory is that parents trade‐off investment in current offspring with investment in future offspring (Stearns 1992). This trade‐off gives rise to sexual conflict over the distribution of parental investment costs (Trivers 1972), which in turn reduces the overall parental investment each offspring receives (Royle et al. 2002b, 2010; Lessells and McNamara 2012). In species that form relatively short‐term pair bonds, breeding partners have little interest in the long‐term reproductive potential of their partner and sexual conflict should be more intense (Lessells and McNamara 2012; Bebbington and Hatchwell 2016); perhaps one reason why begging is less honest in divorce‐prone species is that offspring have to compete more for relatively little parental investment. The interplay between sexual conflict over parental care, parent–offspring conflict, and sibling rivalry is being increasingly recognized as an important source of information about the evolution of family life (Kölliker et al. 2012; Royle et al. 2014, 2016); considering the role of social evolution in the light of multiple levels of conflict will hopefully inspire future studies of offspring begging.

## Concluding Remarks

Interspecific variation in honesty of begging signals is an important source of information to make inferences about how selection acts according to social and ecological circumstances. The frequently hypothesized role of kin selection in mediating parent–offspring conflict (Trivers 1974; Mock and Parker 1997), and thus begging honesty, is intriguing and certainly merits further investigation. However, based on the current evidence, we argue that we lack any firm empirical evidence that kin selection is important in this context. In conclusion, we propose that the results of Caro et al. (2016) demonstrate convincing evidence that scramble competition for limited resources is the main driver of interspecific variation in the honesty of begging signals in birds. Although kin selection is likely to play an important role in the evolution and stability of family life (Emlen 1995), it is crucial that we account for all sources of variation in inclusive fitness to determine the mechanisms by which it acts. We suggest that considering species‐specific ecology and conflict on different family levels may lead to a more balanced insight into the forces that select for begging honesty, including kin selection.

Associate Editor: A. Gardner

Handling Editor: A. Gardner
